# Post-Discharge Treatment Patterns among Patients Treated with Apixaban or Warfarin during Hospitalization for Venous Thromboembolism (VTE)

**DOI:** 10.3390/jcm13123512

**Published:** 2024-06-15

**Authors:** James C. Coons, Vamshi Ruthwik Anupindi, Riddhi Doshi, Mitch DeKoven, Feng Dai, Cristina Russ, Robert Stellhorn, Dong Cheng, Liucheng Shi, Serina Deeba, Dionne M. Hines

**Affiliations:** 1 Department of Pharmacy, UPMC Presbyterian Hospital, Pittsburgh, PA 15213, USA; 2 Department of Pharmacy and Therapeutics, University of Pittsburgh School of Pharmacy, Pittsburgh, PA 15261, USA; 3 IQVIA Inc., Durham, NC 27703, USAmitch.dekoven@iqvia.com (M.D.); liucheng.shi@iqvia.com (L.S.); 4 Pfizer, New York, NY 10001, USA; 5 Bristol Myers Squibb, Lawrenceville, NJ 08543, USA

**Keywords:** apixaban, warfarin, persistence, venous thromboembolism

## Abstract

**Background:** Oral anticoagulants (OACs), such as apixaban and warfarin, are indicated for reducing the risk of recurrent venous thromboembolism (VTE) and are often initiated in the hospital. The aim of this study was to evaluate OAC continuity from inpatient to outpatient settings and the risk of recurrent VTE among patients with an initial event. **Methods:** This retrospective cohort study utilized hospital charge data and medical and prescription claims from 1 July 2016 to 31 December 2022 to identify adults treated with apixaban or warfarin while hospitalized for VTE. Patients were followed to assess switching or discontinuation post-discharge and the risk of recurrent VTE. The index date was the date of the first apixaban or warfarin claim within 30 days post-discharge. **Results:** Of the 19,303 eligible patients hospitalized with VTE, 85% (*n* = 16,401) were treated with apixaban and 15% (*n* = 2902) received warfarin. After discharge, approximately 70% had ≥1 fill for their respective apixaban or warfarin therapy. The cumulative incidence of discontinuation over the 6 months following index was 50.5% and 52.2% for the apixaban and warfarin cohorts, respectively; the cumulative incidence of switching was 6.0% and 20.9%, respectively. The incidence rates of recurrent VTE were 1.2 and 2.5 per 100 person-years for the apixaban and warfarin cohorts, respectively. **Conclusions:** The majority of patients continued their apixaban or warfarin therapy following hospital discharge; however, a considerable proportion either switched or discontinued OAC upon transitioning from inpatient care. Among those who continued therapy, discontinuation, switch, and recurrent VTE occurred less often with apixaban vs. warfarin.

## 1. Introduction

Venous thromboembolism (VTE) is a serious health concern, resulting in approximately 300,000 hospitalizations each year in the United States (US), and includes both deep vein thrombosis (DVT) and pulmonary embolism (PE) [1,2]. VTE can be a recurring event and is fatal for approximately 25% of patients [3,4]. Approximately 30% of patients with a VTE event have a recurrent VTE, most commonly within the first 12 months after the first VTE [2,4,5].

For many years, the mainstay anticoagulant therapy was warfarin, a vitamin K antagonist. Direct oral anticoagulants (DOACs) were first approved by the U.S. Food and Drug Administration for VTE in the 2010s, with rivaroxaban approved in 2012, dabigatran and apixaban in 2014, and edoxaban in 2015. DOACs do not require regular laboratory monitoring and have fewer drug interactions compared to warfarin. The current clinical guidelines from the American Society of Hematology (ASH) suggest using DOACs over warfarin for patients with VTE, citing reduction in the risk of DVT and PE, as well as reducing the risk of adverse events like major bleeding [6].

Anticoagulant therapy, including DOAC and warfarin, is effective in preventing a VTE recurrence, but treatment must be continued to remain effective [7]. ASH guidelines recommend anticoagulant therapy for 3 to 6 months as the primary treatment, with subsequent long-term or extended therapy based on a patient’s risk of recurrence [6,8]. After treating the acute event in an inpatient setting, most VTE patients continue treatment in ambulatory care [9]. It is important that this transition from inpatient to outpatient setting is managed effectively, as it can impact patient outcomes [10]. Only a limited number of studies have investigated such transitions [8,9]; thus, further analysis is warranted to evaluate continuity of treatment following a hospitalization for VTE. 

The goal of this study was to evaluate the treatment patterns (such as treatment discontinuation and switching) that follow a discharge among patients who were hospitalized for a VTE and received apixaban (the most commonly prescribed DOAC) or warfarin therapy during their hospitalization. Additionally, we assessed the risk of recurrent VTE among patients who continued their initial OAC therapy.

## 2. Materials and Methods

### 2.1. Study Design and Data Source

This retrospective, observational study was conducted using linked patient data from IQVIA’s hospital charge data master (CDM), professional medical claims (Dx), and longitudinal prescription claims (LRx) databases from 1 July 2016 to 31 December 2022 (study period). The CDM includes data regarding over 8 million annual inpatient stays from over 450 U.S. hospitals and was used to identify hospitalized patients with a primary diagnosis of VTE and to capture their inpatient treatment. Dx represents about 70% of physician activity in the U.S. from approximately 800,000 office-based physicians and specialists and was used to assess baseline patient characteristics as well as recurrent VTE outcomes. LRx contains information regarding dispensed prescriptions sourced from retail, mail, long-term care, and specialty pharmacies and provides 92% coverage of retail pharmacies across the U.S.; it was used to capture baseline treatment history and post-index treatment patterns. This retrospective study involved secondary analysis of de-identified data and was not considered human subject research, thereby precluding the need for review by an institutional review board.

### 2.2. Population

**Figure 1:**
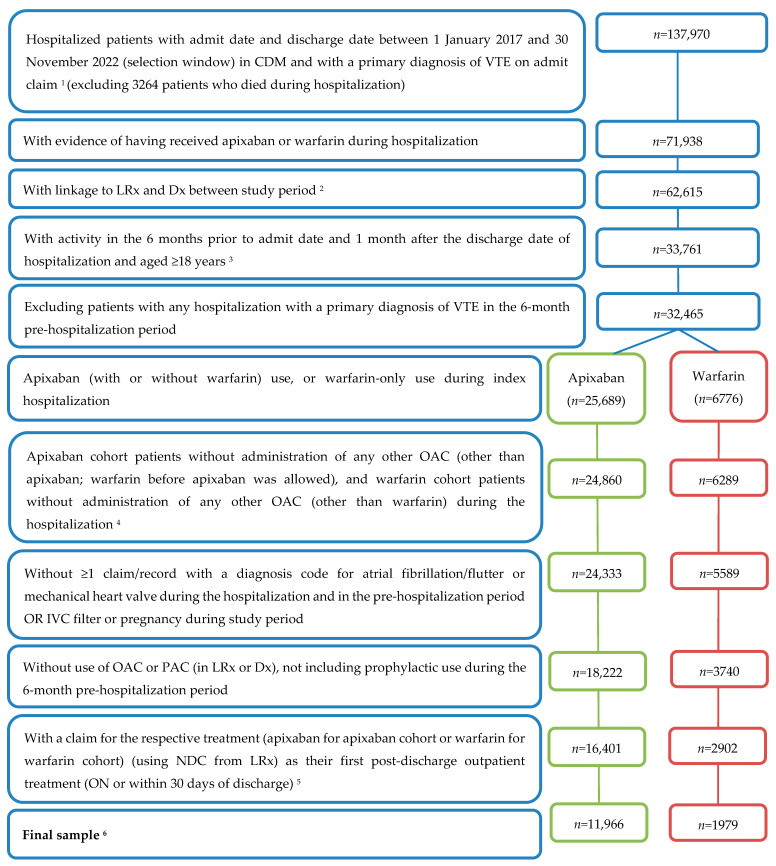
Study Attrition. Abbreviations: IVC: inferior vena cava; OAC: oral anticoagulant; PAC: parenteral anticoagulants; VTE: venous thromboembolism. ^1^ The date of the record with first apixaban or warfarin administration during the index hospitalization was flagged. Patients were assigned to the apixaban or warfarin cohort based on the OAC received on the index date. Patients with prescriptions for both OACs of interest were included in both cohorts at this step. There were 1031 patients with both apixaban and warfarin use during the hospitalization. ^2^ Study period spanned 1 July 2016 to 31 December 2022. ^3^ Activity was defined as ≥1 claim in Dx and ≥1 prescription claim in LRx. ^4^ Patients with OACs other than apixaban or warfarin were also excluded in this step. ^5^ The first post-discharge outpatient prescription claim date in LRx (apixaban or warfarin) was defined as the ‘treatment index date’ for patients in both cohorts, respectively. ^6^ The final sample excluded patients with recurrent VTE (*n* = 7), major bleeding (*n* = 5), or CRNM bleeding (*n* = 1093) events between the discharge date and the treatment index date.

Patients aged ≥18 years with hospital inpatient admission and discharge dates between 1 January 2017 and 30 November 2022 (selection window) and an International Classification of Diseases, Tenth Revision, Clinical Modification (ICD-10-CM) diagnosis code indicative of VTE (including DVT and/or PE) as the primary diagnosis on the admit claim were identified. To be eligible for the hospitalized cohort, patients were required to have medical and pharmacy activity (defined as ≥1 medical claim in Dx and ≥1 prescription claim in LRx) during the 6 months prior to the admit date (pre-hospitalization period) and 1 month (minimum) from the discharge date (post-hospitalization period). To capture patients with an incident hospitalization, patients with previous hospitalizations for VTE during the 6-month pre-hospitalization period were excluded. Similarly, patients with claims for OAC or parenteral anticoagulation use (unless prophylactic) during the 6-month pre-hospitalization period were also excluded to only include patients with incident treatment for VTE. Patients who died during hospitalization (identified from discharge disposition measure) were also excluded. Additional exclusion reasons are outlined in Figure 1.

Based on the OAC received during the VTE hospitalization, patients were classified into one of 2 cohorts: apixaban and warfarin. Patients in the apixaban cohort were those who received apixaban and had no other DOAC during the hospitalization, though warfarin prior to apixaban administration was allowed. Patients in the warfarin cohort had no other OAC during the hospitalization. Patients were required to have their respective apixaban or warfarin treatment on or the day prior to the discharge date, to establish a basis for continued treatment post-discharge. 

Within these initial apixaban and warfarin cohorts, patients with ≥1 outpatient pharmacy claim for the respective index OAC (apixaban for apixaban cohort or warfarin for warfarin cohort) on their discharge date or ≤30 days after discharge (and without claims for any other OAC) were identified as the subset of patients continuing inpatient OAC treatment. The date of the first outpatient apixaban or warfarin claim was termed the treatment index date. Patients with >1 OAC prescription on the treatment index date without pharmacy stability (defined as consistent reporting of data in each month from the pharmacy associated with the treatment index date in LRx) during the 6 months preceding the treatment index date, and with recurrent VTE, major bleeding, or clinically relevant non-major bleeding between the discharge date and the treatment index date were excluded. The subset of patients remaining after applying these criteria were the final apixaban and warfarin cohorts used for the evaluation of study outcomes. Follow-up began on the treatment index date and continued until the end of patient activity (the latter of the last prescription claim for the index therapy or last medical claim), end of pharmacy stability, or end of study period; whichever occurred first. 

### 2.3. Demographic and Clinical Characteristics

VTE diagnosis type (DVT or PE) was characterized based on the VTE diagnosis appearing on the hospital claim. Demographic characteristics, including age, gender, and insurance payer type, were assessed on the treatment index date. Baseline clinical characteristics, including prescribing physician specialty; Charlson comorbidity index (CCI, Dartmouth-Manitoba adaptation) [11]; comorbidities; procedures (orthopedic surgery or placement of central venous catheter); gastrointestinal, intracranial, and other bleeds; history of falls and fracture/trauma of lower extremity; and baseline medication use were assessed during the 6 months prior to the treatment index date. 

### 2.4. Study Outcomes 

Study outcomes included treatment patterns (persistence, discontinuation, and switch) and recurrent VTE. Persistent days was defined as the number of days from the index prescription date until the first of the following: treatment discontinuation, treatment switch, or the end of follow-up. Discontinuation was defined as the occurrence of a ≥30-day gap from the last day of days’ supply of the last fill for the index therapy (prior to the gap) with no other claims for an OAC or to the date of the next claim for a different OAC (thus, discontinuation of apixaban or warfarin included patients who switched to a different OAC or who discontinued OACs completely). Patients were considered to have switched if they filled a prescription for an OAC other than the index apixaban or warfarin, respectively, or received parenteral anticoagulants (PACs), within 30 days before or after the last day of days’ supply of the index treatment. 

Recurrent VTE was evaluated among the apixaban and warfarin cohorts while persistent on their respective therapy. Recurrent VTE was defined as any hospitalization with a primary diagnosis of VTE occurring at least 7 days after the first discharge date and after the treatment index date. 

### 2.5. Statistical Analysis 

Descriptive analysis was performed for all study measures for the final apixaban and warfarin cohorts. Categorical measures were presented using frequencies and percentages, and continuous and count variables were presented as mean, SD, median, minimum, maximum, and interquartile range (IQR). No bivariate comparisons were made between the two cohorts. 

Incidence rates (IR) were presented per 100 person-years with 95% confidence intervals (CI) for recurrent VTE, computed by dividing the number of new events by the person-years at risk (while persistent on therapy). Kaplan Meier curves were generated to report the cumulative incidence of discontinuation, switching, and recurrent VTE at 6 and 12 months post-index for both cohorts. Cox Proportional Hazards models were developed to examine the adjusted risks of discontinuing the index OAC treatment, switching the index OAC treatment, and experiencing a recurrent VTE. The main effect variable for each model was the treatment cohort (apixaban vs. warfarin [warfarin as the reference]). The models included demographic and clinical covariates that were identified based on clinical relevance. A *p*-value of <0.05 was considered statistically significant. All analyses were conducted using SAS^®^ Release 9.4 (SAS Institute Inc., Cary, NC, USA).

## 3. Results

In total, 141,234 hospitalized VTE patients were initially identified. After the application of the inclusion/exclusion criteria, there were 16,401 patients who received apixaban during the hospitalization and 2902 patients who received warfarin. Among those who received apixaban, 341 patients (2.1%) switched to a different OAC on or within the 30 days post-discharge, and 2176 patients (13.3%) had no OAC during this window. Among those with warfarin during their hospitalization, 193 (6.7%) switched to a different OAC and 363 (12.5%) had no OAC within 30 days post-discharge. As depicted in Figure 1, this resulted in 13,945 patients (11,966 apixaban and 1979 warfarin) eligible for the final analytic cohort, representing those who continued their respective therapy in outpatient care. 

### 3.1. Baseline Demographic and Clinical Characteristics

The mean ages of patients in the apixaban and warfarin cohorts were 66.6 (SD: 14.5) and 64.4 (SD: 16.1) years, respectively. Females comprised over 50% of each cohort. Across index years, the proportion of patients in the apixaban cohort increased (63.3% in 2017 to 96.6% in 2021) and the proportion in the warfarin cohort decreased (36.7% in 2017 to 3.4% in 2021).

The majority of patients had an index diagnosis of PE with or without concurrent DVT (apixaban: 82.3%; warfarin: 74.7%). The mean CCI scores for the apixaban and warfarin cohorts were 2.6 and 2.8, respectively. Frequently observed comorbidities were hypertension, hyperlipidemia, diabetes, obesity, and coronary heart disease. The most common baseline medications were angiotensin-converting enzyme inhibitors/angiotensin 2 receptor blockers, statins, and gastroprotective agents (Table 1).

### 3.2. Outcomes

#### 3.2.1. Treatment Patterns

**Figure 2:**
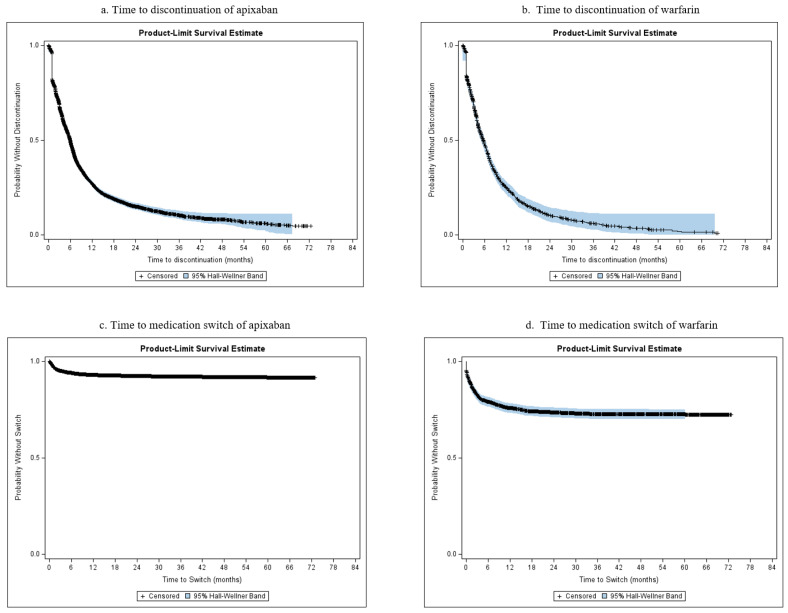
Time to discontinuation and time to switching of apixaban and warfarin cohorts.

Over a mean follow-up of 756.7 days (25.2 months), apixaban patients were persistent for an average of 233.5 (median: 130.0) days. Patients in the warfarin cohort had a mean of 242.6 (median: 139.0) persistent days over a mean follow-up of 1092.8 days (36.4 months). The cumulative incidence of discontinuation at 6 months was 50.5% and 52.2% for the apixaban and warfarin cohorts, respectively. The cumulative incidence of discontinuation at 12 months increased to 72.9% and 75.2% for the apixaban and warfarin cohorts, respectively. Figure 2 depicts the median time to discontinuation from the index treatment, showing 5.97 and 5.67 months for the apixaban and warfarin cohorts, respectively. The cumulative incidence of switching at 6 months was 6.0% and 20.9% for the apixaban and warfarin cohorts, respectively, and at 12 months the cumulative incidence was 7.1% and 24.3% for the apixaban and warfarin cohorts. The median time to switch from the index treatment could not be estimated given that more than half the patients did not experience a switch.

**Figure 3:**
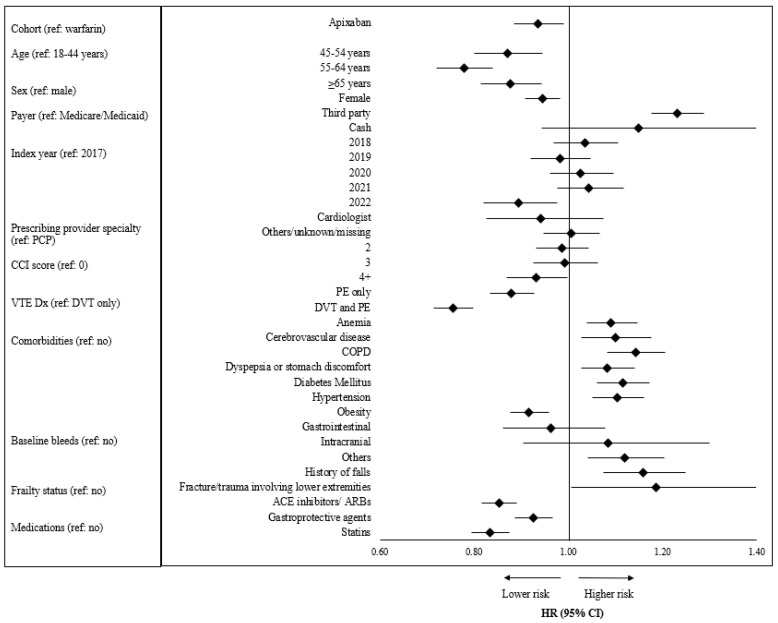
Adjusted risk of discontinuation of apixaban or warfarin. Abbreviations: ACE: angiotensin-converting enzyme; ARB: angiotensin receptor blockers; CCI: Charlson comorbidity index; COPD: chronic obstructive pulmonary disease; DVT: deep vein thrombosis; NSAID: nonsteroidal anti-inflammatory drugs; PCP: primary care physician; PE: pulmonary embolism; VTE: venous thromboembolism.

Figure 3 shows results from the adjusted regression analysis of discontinuation. Patients in the apixaban cohort had a 6.4% significantly lower risk of discontinuing their treatment compared to warfarin patients (Hazard ratio (HR) [95% CI]: 0.94 [0.89–0.99], *p* = 0.02). Patients aged 45–54 years (HR [95% CI]: 0.86 [0.77–0.97]), 55–64 years (HR [95% CI]: 0.77 [0.69–0.86]), and ≥65 years (HR [95% CI]: 0.87 [0.78–0.97]) had a lower risk of discontinuing their treatment compared to patients aged 18–44 years. Patients with a third-party payer had a 23.1% higher risk of discontinuing their treatment compared to those with Medicaid or Medicare (HR [95% CI]: 1.23 [1.18–1.29], *p* < 0.0001). Patients with PE only (HR [95% CI]: 0.88 [0.83–0.93], *p* < 0.0001) and patients with DVT and PE (HR [95% CI]: 0.76 [0.71–0.80], *p* < 0.0001) as their index VTE type had a lower risk of discontinuing their treatment compared to patients with DVT only.

**Figure 4:**
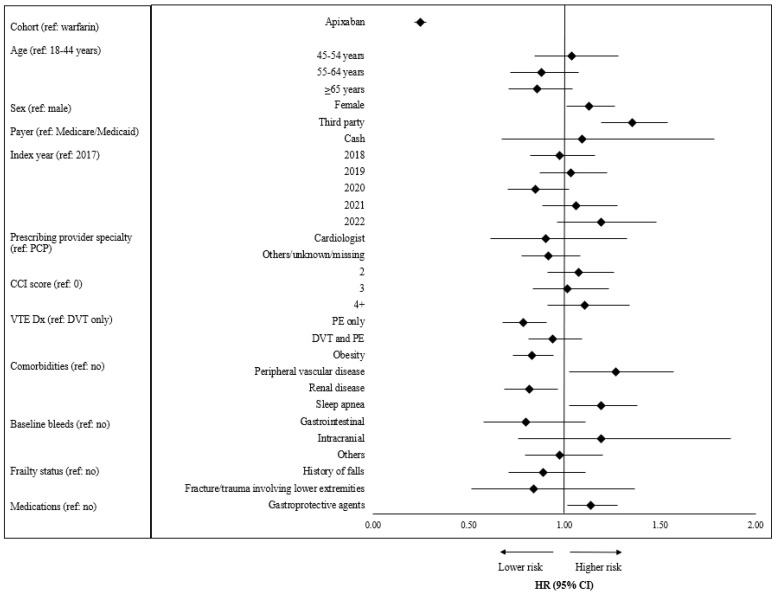
Adjusted risk of switching from apixaban or warfarin. Abbreviations: ACE: angiotensin-converting enzyme; ARB: angiotensin receptor blockers; CCI: Charlson comorbidity index; COPD: chronic obstructive pulmonary disease; DVT: deep vein thrombosis; NSAID: nonsteroidal anti-inflammatory drugs; PCP: primary care physician; PE: pulmonary embolism; VTE: venous thromboembolism.

Figure 4 shows results from the adjusted regression analysis of switching. Patients in the apixaban cohort had a 75.5% significantly lower risk of switching to another OAC compared to the warfarin cohort (HR [95% CI]: 0.25 [0.22–0.28], *p* < 0.0001). Patients with PE only as the index VTE type had a 21.6% lower risk (HR [95% CI]: 0.78 [0.68–0.91], *p* = 0.001) of switching their treatment compared to patients with DVT only. Female patients had a 13.0% higher risk of switching their treatment compared to male patients (HR [95% CI]: 1.13 [1.01–1.26], *p* = 0.0327). Patients with a third-party payer had a 35.5% higher risk of switching their treatment compared to patients with Medicaid or Medicare (HR [95% CI]: 1.36 [1.19–1.54], *p* < 0.0001).

#### 3.2.2. Recurrent VTE

**Figure 5:**
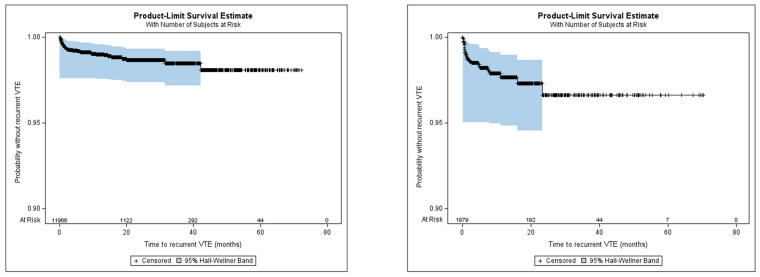
Time to recurrent VTE of apixaban and warfarin cohorts.

The incidence rate of recurrent VTE was 1.2 per 100 person-years in the apixaban cohort and 2.5 per 100 person-years in the warfarin cohort, depicted in Figure 5. The cumulative incidence of recurrent VTE at 6 months was 0.8% and 1.8% for the apixaban and warfarin cohorts, respectively. The cumulative incidence of recurrent VTE at 12 months was 1.0% and 2.3% for the apixaban and warfarin cohorts, respectively. Because recurrent VTE occurred in <50% of the patients, the median time to event could not be estimated.

**Figure 6:**
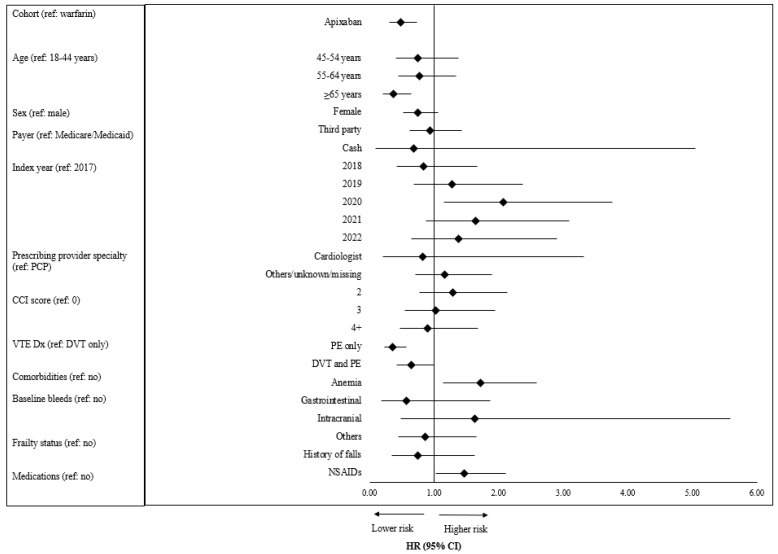
Adjusted risk of recurrent VTE among VTE patients treated with apixaban or warfarin. Abbreviations: CCI: Charlson comorbidity index; PCP: primary care physician; VTE: venous thromboembolism; DVT: deep vein thrombosis; PE: pulmonary embolism; NSAIDs: nonsteroidal anti-inflammatory drugs.

Patients in the apixaban cohort had a 52.4% significantly lower risk of recurrent VTE compared to those in the warfarin cohort (HR: 0.48; 95% CI: 0.31–0.74, *p* = 0.001), depicted in Figure 6. Patients with PE only as the index VTE type had a 64.2% lower risk (HR [95% CI]: 0.36 [0.26–0.57], *p* < 0.0001) of recurrent VTE compared to patients with DVT only.

## 4. Discussion

OAC therapy is a standard treatment for VTE that may be initiated in the hospital. Guidelines recommend that following the initial management of VTE, patients should continue therapy for at least 3 to 6 months to reduce the risk of recurrence [6]. In this analysis, we evaluated continuity of OAC therapy post-discharge among patients treated with apixaban or warfarin while hospitalized for VTE. Among the 19,303 patients we identified that were hospitalized for VTE and treated with apixaban (85.0%) or warfarin (15.0%), 2.8% overall had a switch to an OAC other than the initial apixaban or warfarin therapy, and 13.2% overall did not have an OAC within the 30 days following discharge. A prior VTE study attempted to evaluate treatment patterns as patients transitioned from inpatient to outpatient care; however, their reporting was hindered by the lack of data that followed patients across settings [12]. Burton et al. used electronic health records linked to claims data to also evaluate treatment patterns during care transition, though their analysis was constrained by small sample sizes. Nevertheless, the authors observed that 40% of inpatients with VTE did not have anticoagulation treatment post-discharge [8]. While our finding of 13.2% overall without an OAC indicates an improvement in treatment continuity, it highlights the need for continued efforts in optimally managing VTE and coordinating therapy.

Notably, the majority of patients within our cohort continued their respective apixaban or warfarin therapy following hospital discharge. Further evaluation of these patients suggests the need for initiatives that enhance treatment persistence, as median persistence with apixaban or warfarin was approximately 6 months for both cohorts. This finding is consistent with that of Iyer et al., who utilized Medicare Fee-for-Service and commercial healthcare claims data to identify patients with VTE who initiated DOACs within 90 days following hospitalization. Their analysis showed that patients within their study were persistent on treatment for approximately 6 months (Medicare: median, 175 days [IQR, 76–327 days]; commercial insurance: median, 168 days [IQR, 83–279 days]) [13]. In a separate analysis of patients with VTE within an integrated healthcare delivery system, Packard et al. found that 68.2% of patients treated with a DOAC were persistent beyond 6 months [7]. Whereas the duration of OAC therapy for VTE is driven by many factors, such as VTE etiology (provoked vs. unprovoked) or the presence of cancer-related thrombosis, these observations imply that many patients are not treated beyond the primary treatment phase. Reasons for treatment discontinuation or switching were not evaluated in the current study; however, we found that patients on apixaban had a significantly lower risk of discontinuing or switching medication compared to patients on warfarin. 

A critical aspect of OAC treatment continuity is recurrent VTE risk reduction. We found that during the time persistent with therapy, patients treated with apixaban had a 52.1% lower risk of recurrent VTE compared to patients treated with warfarin. Other studies have reported similar observations, including one that compared the risk of recurrent VTE among Medicare enrollees with the use of an OAC for at least 90 days post-hospitalization for VTE. Results from that analysis showed a 31% lower risk of hospitalization for recurrent VTE in patients treated with apixaban compared to warfarin [14]. Likewise, Park et al. evaluated Medicare enrollees with VTE and reported an 87% lower risk of recurrent VTE with the use of apixaban vs. warfarin [15]. A comparative study among patients with VTE with commercial and Medicare Supplemental health plans also demonstrated a significantly lower risk of recurrent VTE (46%) among patients with apixaban compared to warfarin [16]. Considering the evidence provided by these studies and the present one, the association of a greater reduction in risk of recurrent VTE with apixaban compared to warfarin can be seen across various populations. 

The findings from this study should be interpreted with consideration of certain limitations. Claims and hospital charge data are gathered for medical billing purposes and not research; therefore, there is potential for miscoding or misclassification, and the claims may not reflect true diagnoses or treatment. Further, given the open-source nature of the databases used in this study, the data may not be comprehensive, with only information from the pharmacies, offices, and hospitals that contribute to the databases being captured. Nonetheless, LRx has high prescription coverage (approximately 92% of retail pharmacies in the U.S.) and includes claims across all payers as well as self-pay, rendering it suitable for treatment pattern analysis such as that conducted here. We evaluated treatment continuity in this study; however, we were not able to capture medications provided as samples, thus our results may be underestimated. Additionally, the presence of a claim for medication does not indicate that the medication was consumed nor that it was taken as prescribed.

Results from retrospective studies are subject to confounding and can only establish associations; causal relationships should not be inferred and can only be determined by clinical trials. Not all variables of importance are available in real-world datasets, so there could be differences in study cohorts resulting in potential residual confounding. Finally, we followed patients who initiated treatment in the hospital to determine their outpatient treatment patterns; the results presented here may not be generalizable to patients who initiate therapy in an outpatient setting, as well as those who are not transitioning care.

## 5. Conclusions

Though some patients had a disruption in OAC therapy (in the form of treatment switching or discontinuation) as they transitioned from inpatient to outpatient VTE care, the majority of patients did continue their therapy. However, treatment was generally short-term, with longer durations for patients on apixaban than warfarin. Our study highlights the reduced risk of VTE recurrence with continued treatment, especially with DOACs such as apixaban. A better understanding of the factors that promote prolonged persistence with OACs among patients with VTE is warranted.

## Figures and Tables

**Table 1:** Patient demographic and clinical characteristics.

	Apixaban *n* = 11,966	Warfarin *n* = 1979
**Age (years; mean, SD)**	66.6 (14.5)	64.4 (16.1)
**Age group (years; *n*, %)**		
18–44	1009 (8.4%)	253 (12.8%)
45–54	1256 (10.5%)	268 (13.5%)
55–64	2472 (20.7%)	357 (18.0%)
≥65	7229 (60.4%)	1101 (55.6%)
**Sex (*n*, %)**		
Male	5252 (43.9%)	849 (42.9%)
Female	6714 (56.1%)	1130 (57.1%)
**Index year (*n*, %)**		
2017	1330 (11.1%)	772 (39.0%)
2018	1677 (14.0%)	424 (21.4%)
2019	2172 (18.2%)	349 (17.6%)
2020	2296 (19.2%)	225 (11.4%)
2021	2575 (21.5%)	142 (7.2%)
2022	1916 (6.0%)	67 (3.4%)
**Follow-up duration (months; mean, SD)**	25.2 (18.9)	36.4 (22.0)
**Prescribing physician specialty (*n*, %)**		
PCP	10,336 (86.4%)	1664 (84.1%)
Cardiologist	266 (2.2%)	44 (2.2%)
Others/unknown/missing	1364 (11.4%)	271 (13.7%)
**CCI score (*n*, %)**		
0–1	4987 (41.7%)	763 (38.6%)
2	2039 (17.0%)	339 (17.1%)
3	1485 (12.4%)	232 (11.7%)
4+	3455 (28.9%)	645 (32.6%)
**Index VTE type (*n*, %)**		
DVT only	2119 (17.7%)	500 (25.3%)
PE only	5696 (47.6%)	850 (43.0%)
DVT and PE	4151 (34.7%)	629 (31.8%)
**Comorbidities (*n*, %)**		
Anemia	2946 (24.6%)	575 (29.1%)
Cerebrovascular disease	1364 (11.4%)	261 (13.2%)
Coronary heart/ischemic heart disease	3657 (30.6%)	630 (31.8%)
Congestive heart failure	1996 (16.7%)	363 (18.3%)
COPD	2169 (18.1%)	350 (17.7%)
Dementia/Alzheimer’s disease	868 (7.3%)	149 (7.5%)
Dyspepsia	2074 (17.3%)	383 (19.4%)
Diabetes mellitus	3343 (27.9%)	653 (33.0%)
Hyperlipidemia	4903 (41.0%)	815 (41.2%)
Hypertension	8108 (67.8%)	1385 (70.0%)
Liver disease	1058 (8.8%)	177 (8.9%)
Gall bladder/Pancreatic disease	781 (6.5%)	127 (6.4%)
Obesity	3580 (29.9%)	758 (38.3%)
Peripheral vascular disease	691 (5.8%)	146 (7.4%)
Pneumonia	1815 (15.2%)	282 (14.2%)
Renal disease	2310 (19.3%)	511 (25.8%)
Sleep apnea	1720 (14.4%)	369 (18.6%)
**Procedures of interest (*n*, %)**		
Orthopedic surgery	583 (4.9%)	140 (7.1%)
Central venous catheter	1010 (8.4%)	196 (9.9%)
**Baseline bleeds (*n*, %)**		
Gastrointestinal	353 (3.0%)	89 (4.5%)
Intracranial	130 (1.1%)	40 (2.0%)
Others	1052 (8.8%)	179 (9.0%)
**History of falls (*n*, %)**	924 (7.7%)	176 (8.9%)
**Fracture/trauma involving lower extremities (*n*, %)**	158 (1.3%)	48 (2.4%)
**Medications of interest (*n*, %) (>5%)**		
ACE inhibitors/ARBs	4854 (40.6%)	798 (40.3%)
Antiarrhythmic agents	920 (7.7%)	214 (10.8%)
Antiplatelet agents	692 (5.8%)	127 (6.4%)
Beta blockers	3487 (29.1%)	623 (31.5%)
Gastroprotective agents	4234 (35.4%)	731 (36.9%)
NSAIDs	4082 (34.1%)	661 (33.4%)
Statins	4437 (37.1%)	709 (35.8%)

Abbreviations: SD: standard deviation; ACE: angiotensin-converting enzyme; ARB: angiotensin receptor blockers; CCI: Charlson comorbidity index; COPD: chronic obstructive pulmonary disease; DVT: deep vein thrombosis; NSAIDs: nonsteroidal anti-inflammatory drugs; PCP: primary care physician; PE: pulmonary embolism.

## Data Availability

Restrictions apply to the availability of these data. Data were obtained from IQVIA and are available from IQVIA through a license agreement.

## Abbreviations

ASH: American Society of Hematology; CCI: Charlson comorbidity index; CI: confidence interval; DOAC: direct oral anticoagulant; DVT: deep vein thrombosis; HR: hazard ratio; IQR: interquartile ratio; IR: incidence rate; OAC: oral anticoagulant; PE: pulmonary embolism; U.S.: United States; VTE: venous thromboembolism.

## References

[1] Agency for Healthcare Research and Quality. Healthcare Cost and Utilization Project (HCUP): HCUP Databases. National (Nationwide) Inpatient Sample (NIS) Database Documentation. https://hcup-us.ahrq.gov/db/nation/nis/nisdbdocumentation.jsp.

[2] Heit J.A. (2015). Epidemiology of venous thromboembolism. Nat. Rev. Cardiol..

[3] Tagalakis V., Patenaude V., Kahn S.R., Suissa S. (2013). Incidence of and mortality from venous thromboembolism in a real-world population: The Q-VTE Study Cohort. Am. J. Med..

[4] Beckman M.G., Hooper W.C., Critchley S.E., Ortel T.L. (2010). Venous thromboembolism: A public health concern. Am. J. Prev. Med..

[5] Centers for Disease Control and Prevention Venous Thromboembolism (Blood Clots). https://www.cdc.gov/blood-clots/data-research/facts-stats/index.html.

[6] Ortel T.L., Neumann I., Ageno W., Beyth R., Clark N.P., Cuker A., Hutten B.A., Jaff M.R., Manja V., Schulman S. (2020). American Society of Hematology 2020 guidelines for management of venous thromboembolism: Treatment of deep vein thrombosis and pulmonary embolism. Blood Adv..

[7] Packard A., Delate T., Martinez K., Clark N.P. (2020). Adherence to and persistence with direct oral anticoagulant therapy among patients with new onset venous thromboembolism receiving extended anticoagulant therapy and followed by a centralized anticoagulation service. Thromb. Res..

[8] Burton T., Hlavacek P., Guo J.D., Rosenblatt L., Mardekian J., Ferri M., Russ C., Kline J.A. (2020). Clinical characteristics and treatment patterns of patients with venous thromboembolism (VTE) transitioning from hospital to post-discharge settings. Hosp. Pract..

[9] Lenchus J.D. (2016). Transitions in the Prophylaxis, Treatment and Care of Patients with Venous Thromboembolism. Adv. Ther..

[10] Brock J., Mitchell J., Irby K., Stevens B., Archibald T., Goroski A., Lynn J. (2013). Association between quality improvement for care transitions in communities and rehospitalizations among Medicare beneficiaries. JAMA.

[11] Romano P.S., Roos L.L., Jollis J.G. (1993). Adapting a clinical comorbidity index for use with ICD-9-CM administrative data: Differing perspectives. J. Clin. Epidemiol..

[12] Trocio J., Rosen V.M., Gupta A., Dina O., Vo L., Hlavacek P., Rosenblatt L. (2019). Systematic literature review of treatment patterns for venous thromboembolism patients during transitions from inpatient to post-discharge settings. Clin. Outcomes Res..

[13] Iyer G.S., Tesfaye H., Khan N.F., Zakoul H., Bykov K. (2023). Trends in the Use of Oral Anticoagulants for Adults with Venous Thromboembolism in the US, 2010–2020. JAMA Netw. Open.

[14] Pawar A., Gagne J.J., Gopalakrishnan C., Iyer G., Tesfaye H., Brill G., Chin K., Bykov K. (2022). Association of Type of Oral Anticoagulant Dispensed with Adverse Clinical Outcomes in Patients Extending Anticoagulation Therapy Beyond 90 Days After Hospitalization for Venous Thromboembolism. JAMA.

[15] Park H., Kang H.-R., Huang P.-L., Lo-Ciganic W.-H., DeRemer C.E., Wilson D., Dietrich E.A. (2023). Comparative effectiveness and safety of extended anticoagulant therapy among Medicare beneficiaries with venous thromboembolism. Clin. Transl. Sci..

[16] Dawwas G.K., Smith S.M., Dietrich E., Lo-Ciganic W.H., Park H. (2020). Comparative effectiveness and safety of apixaban versus warfarin in patients with venous thromboembolism. Am. J. Health Syst. Pharm..

